# Spontaneous isolated brachiocephalic artery dissection with subsequent type-A acute aortic dissection

**DOI:** 10.1093/ehjcr/ytaf142

**Published:** 2025-03-25

**Authors:** Hisato Takagi

**Affiliations:** Department of Cardiovascular Surgery, Shizuoka Medical Center, 762-1 Nagasawa, Shimizu-cho, Sunto-gun, Shizuoka 411-8611, Japan

A 66-year-old woman with neither relevant co-morbidity except for treated hypertension nor history of trauma suffered sudden right neck pain radiating to the right upper extremity. Contrast-enhanced CT scans revealed limited dissection from the brachiocephalic aretry (BCA) (*Figure 1A* and *B*, black arrows) to the right common-carotid (*Figure 1A*, white arrow) and subclavian artery (*Figure 1B*, white arrow) with the patent false lumen. There was no dissection in the ascending, transverse, or descending-thoracic aorta. Spontaneous isolated BCA dissection (BCAD) was diagnosed, and the patient underwent conservative medical (antihypertensive and analgesic) treatment. No genetic testing was performed because the patient presented no physical characteristics of connective tissue disorders such as Marfan, Ehlers–Danlos, or Loeys–Dietz syndrome. Three years later, the patient suffered sudden chest pain, and type-A acute aortic dissection (AD) (TA-AAD) was diagnosed on contrast-enhanced CT scans (*Figure 1C*, white arrows). The false lumen of the AD (*Figure 1D*, white arrow) was not continued to that of the antecedent BCAD (*Figure 1D*, black arrow). Urgent ascending aortic replacement was successfully performed. Aortic arch replacement was not considered because previously-dissected BCA was not enlarged and no aortic entry was intraoperatively identified in the ascending or transverse aorta. A histologic examination of the resected aorta merely demonstrated slight medial myxoid changes. The postoperative course was uneventful. Repeated CT scans during the following 6 years indicated no significant change of the BCAD and the residual AD. Several patients with traumatic BCAD have been often reported (Supplementary References S1–S6). Spontaneous isolated BCAD, however, is extremely rare. Merely 12 cases^1–3^ (Supplementary References S7–S14) including the preset patient (see Supplementary material online, *Table S1*) were identified. Subsequent TA-AAD and preceding type-B acute AD occurred in three patients (including the present case)^1,2^ and one patient,^3^ respectively; i.e. a total of four patients (33.3%) complicated metachronous AD, which suggests some common aetiology between isolated BCAD and AD.

**Figure 1:**
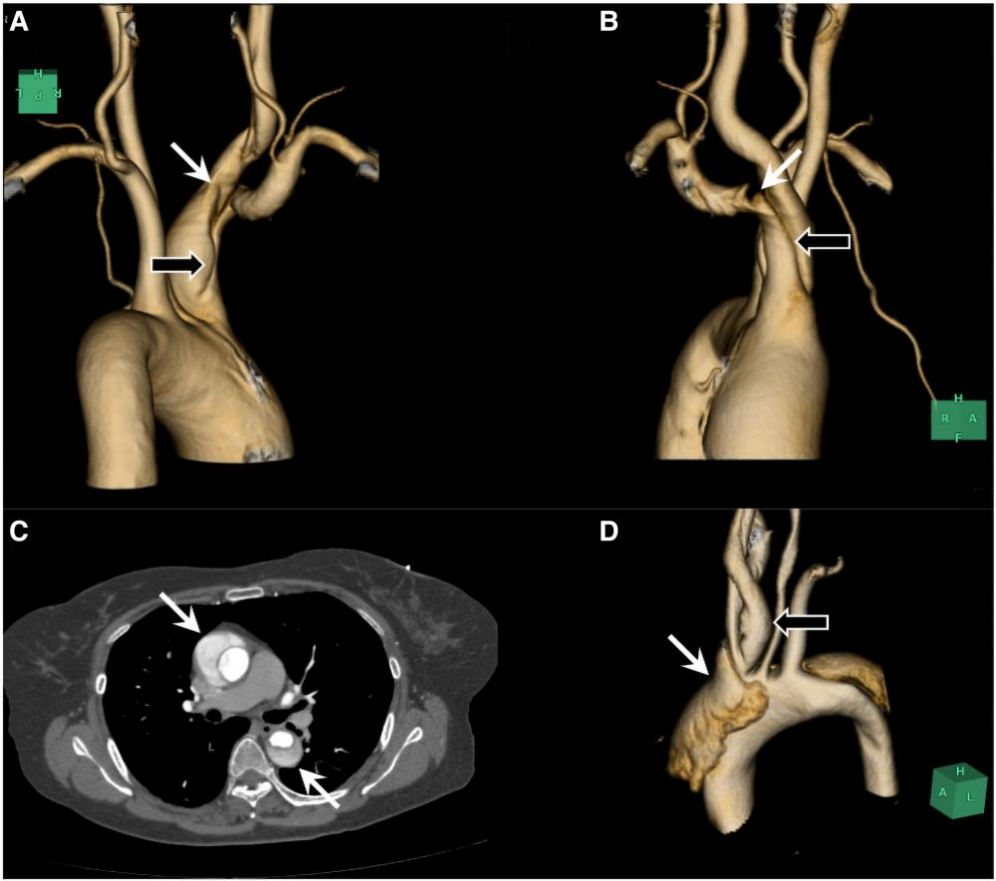
(*A* and *B*) Limited dissection from the BCA (black arrows) to the right common-carotid (*A*, posterior view; white arrow) and subclavian artery (*B*, right-anterior oblique view; white arrow) with the patent false lumen. (*C*) TA-AAD (arrows) 3 years later. (*D*) The false lumen of the AD (white arrow) was not continued to that of the antecedent BCAD (black arrow).

## Supplementary Material

ytaf142_Supplementary_Data

## Data Availability

The data underlying this article are available in the article.

## Notes

**Consent:** The author confirms that written consent for submission and publication of this case report, including images and associated text, has been obtained from the patient in line with COPE guidance.

**Funding**: None declared.
