# Supplementary material for: Spontaneous isolated brachiocephalic artery dissection with subsequent type-A acute aortic dissection
Source: Eur Heart J Case Rep. 2025 Mar 25;9(4):ytaf142. doi: 10.1093/ehjcr/ytaf142 (PMC11969333; doi:10.1093/ehjcr/ytaf142)
Supplement: ytaf142_Supplementary_Data [file ytaf142_supplementary_data.pdf]

Supplementary Table S1 **Reported cases of spontaneous isolated brachiocephalic artery dissection**

Reference	Author reported year	Age (years)	Sex	BCA dissection			Symptoms	Treatment	Prognosis
				Entry	Extension	False lumen			
–	Takagi 2025 (present case)	66	Female	Unknown	RCCA and RSCA	Patent	Right neck pain and migratory pain from right upper extremity to back	Conservative medical (antihypertensive and analgesic) treatment	Alive and survived surgery for TA-AAD 3 years and 2 months later
1	Oura 2022	56	Male	Immediately proximal segment of RICA	RCCA and RICA	Patent	Sudden chest pain and transient left paresthesia	Aspirin	Died of ruptured TA-AAD 18 days later
S7	Kotoulas 2021	71	Female	1-2 mm far away from IA origin	Limited to BCA	Patent	Right cervical pain and ipsilateral arm hypoesthesia	BCA graft reconstruction from ascending aorta and patch repair of aortic arch roof for differential diagnosis of BCA pseudoaneurysm	Alive without additional events 30 months later
S8	Sharaf 2021	50	Male	Unknown	RSCA and RSCA~RAxA	Patent	Shortness of breath and sudden-onset, intermittent, substernal chest pain radiating to both sides of jaw and to right arm	Anti-hypertensive therapy and aspirin	Alive without additional events 1 year later
S9	Seese 2019	68	Male	Unknown	RCCA and RSCA	Patent in BCA and RSCA (thrombosed in RCCA)	Right-sided neck and chest pain	Anti-hypertensive therapy, warfarin, and aspirin	Alive without additional events 6 months later
S10	Nagata 2017	58	Male	Unknown	Limited to BCA	Patent	Back pain	Anti-hypertensive and pain therapy	Alive without additional events 6 months later
S11	Mani 2015	41	Male	Unknown	RCCA	Patent	Ataxia, slurred speech and left facial weakness	Heparin and warfarin	Discharged 4 days later
S12	Ohki 2014	63	Female	Common trunk at BCA origin	LCCA (bovine aortic arch)	Patent	Gradually enlarging right neck pulsatile mass and neck pain (history of severe chest pain 1year before)	Total aortic arch replacement with aneurysmectomy (30-mm BCA aneurysm)	Discharged on 20th postoperative day
S13	Sansone 2013	67	Male	Unknown	Limited to BCA	Patent	Left hemiparesis and dysarthria	Anti-hypertensive therapy (and rt-PA for stroke)	Discharged about 10 days later
S14	Lampropoulos 2009	44	Male	Unknown	RCCA	Patent	Acute, excruciating right shoulder pain with radiation to precordial area and neck and temporary syncopal attack with urine loss	(Died before surgery)	Died of ruptured BCA dissection immediately
2	Munakata 2008	61	Male	Unknown	RCCA	Patent	Sudden onset of right back pain	Reconstruction of RCCA and RSCA from ascending aorta with bifurcated graft for ruptured BCA dissection	Alive and survived surgery for TA-AAD 2 weeks later

3	Kanady 1990	54	Female	BCA origin	Limited to BCA	Thrombosed (partially patent at BCA origin)	Bilateral ear pain progressing to encompass entire right-sided face and neck; and intermittent blurred vison and right-sided facial and tongue numbness	IV anticoagulant therapy and warfarin (surgery for TB-AAD 3 years before)	Alive without additional events 1 year later
	Total	58.3±9.6	Male, 66.7%	–	Limited to BCA, 33.3%	Thrombosed, 8.3%	Neurological symptoms, 50.0%	Surgery, 25.0%	Mortality, 16.7%; subsequent TA-AAD, 25.0% (precedent TB-AAD, 8.3%)

BCA, brachiocephalic artery; LCCA, left subclavian artery; RAxA, right axillary artery; RCCA, right common carotid artery; RICA, right internal carotid artery; RSCA, right subclavian artery; rt-PA, recombinant tissue plasminogen activator; TA-AAD, type-A acute aortic dissection; TB-AAD, type-B acute aortic dissection.

Supplementary References

S1. Deshpande AA, Agasty S, Kumar S, Ramakrishnan P. Isolated traumatic innominate artery dissection: an exceedingly rare entity! *BMJ Case Rep* 2021;**14**:e241710.

S2. Niclauss L, Namasivayam J, Kirsch M, Prêtre R. Traumatic brachiocephalic trunk dissection of a bovine arch. *Eur J Cardiothorac Surg* 2020;**57**:801–802.

S3. Howe KL, Guirgis M, Woodman G, et al. Blunt innominate artery trauma requiring repair and carotid ligation. *Trauma Case Rep* 2017;12:24-7.

S4. Dias-Neto M, Ramos JF, Teixeira JF. Blunt Injury of the Innominate Artery: What Surprises to Expect? A Case Report. *Vasc Endovascular Surg* 2018;**52**:226–232.

S5. Azarcon F, Ghaleb M. Early diagnosis and treatment of a posttraumatic pseudoaneurysm/dissection of the innominate artery. *Prehosp Disaster Med* 2014;**29**:209–211.

S6. Knosalla C, Pasic M, Hetzer R. Traumatic dissection of the innominate artery. *Eur J Cardiothorac Surg* 2000;**18**:370.

S7. Kotoulas C, Georgiou C, Chouliaras E, Theodosiadis N, Kotoulas S, Kotoulas A, et al. Diagnostic dilemma in spontaneous innominate artery pathology: a case report. *Gen Thorac Cardiovasc Surg* 2021;**69**:367–370.S2.

S8. Sharaf OM, Martin TD, Jeng EI. Management of a spontaneous supra-aortic arterial dissection: a case report. *J Med Case Rep* 2021;**15**:283.

S9. Seese L, Kilic A, Navid F, Gleason TG, Sultan I. Nonoperative Management of Brachiocephalic Artery Dissection. *Ann Thorac Surg* 2019;**107**:e97–e9.

S10. Nagata T, Johno H, Wang Y, Asanuma M. Isolated innominate artery dissection. *J Card Surg* 2017;**32**:710–711.

S11. Mani H, Ahluwalia S. Isolated brachiocephalic artery dissection presenting as acute stroke. *BMJ Case Rep* 2015;**2015**:bcr2014208815.

S12. Ohki S, Obayashi T, Koyano T, Yasuhara K, Hirai H, Hatori K. Spontaneous innominate and left common carotid artery dissection with bovine aortic arch. *Gen Thorac Cardiovasc Surg* 2014;**62**:238–240.

S13. Sansone T, Baroncelli S, Tozzetti C, Berni A, Torri M. Challenges in management of ischemic stroke secondary to brachiocephalic artery dissection. *Intern Emerg Med* 2013;**8**:181–183.

S14. Lampropoulos S, Theofilogiannakos EK, Gkontopoulos A, Kadoglou NP, Mamalis V, Kontaki T, et al. Syncope and cardiovocal syndrome as the result of a spontaneous innominate artery dissection. *J Cardiovasc Med (Hagerstown)* 2009;**10**:815–817.
